# The Impact of Long COVID on the Quality of Life

**DOI:** 10.3390/medicina60081359

**Published:** 2024-08-21

**Authors:** Angela Cozma, Adela-Viviana Sitar-Tăut, Olga Hilda Orășan, Daniel Corneliu Leucuța, Tinca-Codruța Pocol, Octavia Sălăgean, Camil Crișan, Nicolae-Dan Sporiș, Andrada-Luciana Lazar, Toma-Vlad Mălinescu, Andreea-Maria Ganea, Călin Vasile Vlad, Melinda Horvat, Mihaela Sorina Lupșe, Violeta Briciu

**Affiliations:** 1 Department of Internal Medicine, “Iuliu Hațieganu” University of Medicine and Pharmacy, 400012 Cluj-Napoca, Romania; angelacozma@yahoo.com (A.C.); adelasitar@yahoo.com (A.-V.S.-T.); tinca_codruta@yahoo.com (T.-C.P.); runcan.octavia@yahoo.ro (O.S.); crisancamil@gmail.com (C.C.); vladvasilecalincfcluj@gmail.com (C.V.V.); 2 Department of Medical Informatics and Biostatistics, “Iuliu Haţieganu” University of Medicine and Pharmacy, 400349 Cluj-Napoca, Romania; danny.ldc@gmail.com; 3 Department of Medical Oncology, Prof. Dr. I. Chiricuța Oncology Institute, 400015 Cluj-Napoca, Romania; 4 Department of Dermatology, “Iuliu Hațieganu” University of Medicine and Pharmacy, 400012 Cluj-Napoca, Romania; andradalazarluciana@yahoo.com; 5 Department of Cardiology, “Iuliu Hațieganu” University of Medicine and Pharmacy, 400012 Cluj-Napoca, Romania; vladd_malinescu@yahoo.com (T.-V.M.); andreeaganeaa@gmail.com (A.-M.G.); 6 Department of Infectious Diseases and Epidemiology, “Iuliu Hațieganu” University of Medicine and Pharmacy, 400348 Cluj-Napoca, Romania; melinda.horvat@umfcluj.ro (M.H.); mihaela.lupse@yahoo.com (M.S.L.); briciu.tincuta@umfcluj.ro (V.B.)

**Keywords:** long COVID, quality of life, omicron variant

## Abstract

*Background and Objectives*: The term long COVID refers to patients with a history of confirmed COVID-19 infection, who present symptoms that last for at least 2 months and cannot be explained by another diagnosis. Objectives: The present study aims to determine the most common symptoms of the long COVID syndrome and their impact on the quality of life. *Materials and Methods*: A prospective observational study was conducted on patients diagnosed with mild and moderate COVID-19 (based on a positive SARS-CoV-2 molecular diagnostic or rapid antigen test and severity form definition) at the Clinical Hospital of Infectious Diseases, Cluj-Napoca, Romania. Clinical examinations with detailed questions about symptoms were performed at the time of the diagnosis of COVID-19 and the six-month follow-up. Two years after COVID-19 infection, patients were invited to complete an online quality-of-life questionnaire regarding long COVID symptoms. *Results*: A total of 103 patients (35.92% males) with a mean age of 41.56 ± 11.77 were included in this study. Of the total number of patients, 65.04% presented mild forms of COVID-19. Data regarding the vaccination status showed that 83.5% were vaccinated against SARS-CoV-2. The most common symptoms at diagnosis were cough (80.6%), fatigue (80.4%), odynophagia (76.7%), and headaches (67.6%), with female patients being statistically more likely to experience it (*p* = 0.014). Patients with moderate forms of the disease had higher levels of both systolic (*p* = 0.008) and diastolic (*p* = 0.037) blood pressure at diagnosis, but no statistical difference was observed in the 6-month follow-up. The most common symptoms at 2 years (in 29 respondent subjects) were represented by asthenia (51.7%), headache (34.5%), memory disorders (27.6%), abdominal meteorism (27.6%), and arthralgia (27.6%). In terms of cardiovascular symptoms, fluctuating blood pressure values (20.7%), palpitations (17.2%), and increased heart rate values (17.2%) were recorded. *Conclusions*: If at the time of diagnosis, the most frequent manifestations of the disease were respiratory, together with headache and fatigue, at re-evaluation, asthenia, decreased effort tolerance, and neuropsychiatric symptoms prevailed. Regarding the cardiovascular changes as part of the long COVID clinical picture, some patients developed prehypertension, palpitations, and tachycardia.

## 1. Introduction

Infection with the SARS-CoV-2 virus represented and still represents a challenge for the medical system, in terms of diagnostics (even in modern times, with artificial intelligence techniques’ development), treatment, and mortality and morbidity [1]. Clinical manifestations of COVID-19 infection can vary from asymptomatic subclinical infections to severe forms of pneumonia that can progress to acute respiratory distress syndrome [2]. Regarding extrapulmonary manifestations, existing evidence has shown hepatic, gastrointestinal, and cardiac involvement. In the case of mild forms of the disease, full recovery is achieved in approximately 7 days, and in severe cases, recovery can reach up to 6–7 weeks. However, more cases are reported in which patients remained symptomatic for a long time, regardless of the severity of the COVID-19 infection, a phenomenon labeled as long COVID [3]. The term long COVID refers to a condition in which patients with a history of confirmed COVID-19 present symptoms that last for at least 2 months and cannot be explained by another diagnosis. It is assumed that the occurrence of the long COVID syndrome is due to the dysfunction of the sympathetic and parasympathetic nervous system [4]. 

The most common acute cardiac complications developed through SARS-CoV-2 infection are represented by arrhythmias, the most common forms being atrial fibrillation and paroxysmal ventricular tachycardia, which account for 10,4% of patients with a moderate or severe form of the disease. Cardiac failure manifested in a variable percentage of patients (between 2.8 and 24%), while acute coronary syndrome was diagnosed in 1.3% to 4.9% of infected patients [5].

Also, recent studies have shown an increase in the incidence of cardiovascular pathologies following the COVID-19 infection, occurring between 30 days and 1 year after infection, with the most common forms represented by arrhythmias (atrial fibrillation or ventricular tachycardia), ischemic coronary diseases, heart failure, thromboembolism, and arterial hypertension [6]. Cardiac complications of long COVID are caused primarily by the alteration in cardiovascular physiology and lesions caused by the myocardial injury during the acute phase [7]. The most common cardiovascular symptoms seen in long COVID are palpitations, dyspnea, syncope, and chest pain [8]. 

Long COVID is also associated with neurological and cognitive symptoms such as cognitive impairment, anxiety and depression, dysgeusia, hyposmia, memory disorders, balance disorders, tinnitus, vertigo, hearing loss, or insomnia [9]. 

Wong et al. demonstrated in a systemic review published in 2021 that myalgic encephalomyelitis and chronic fatigue syndrome consisting of fatigue, reduced daily activity, and post-exertional malaise are encountered very often in the long COVID syndrome, with the most frequent symptom being fatigue [10]. Evcik noted in a study published in 2023 that patients can experience myalgia, arthralgia, decreased ability to exercise, and generalized pain [11].

In a study published in 2021, Ding et al. emphasized a correlation between COVID-19 and ovarian injury including decreased ovarian reserve and other various hormonal disorders, with the most frequent clinical manifestation being changes in the menstrual cycle [12]. 

Regarding respiratory symptoms, dyspnea and cough are the most frequent symptoms described by Davis et al. in a study published in 2021 [9]. Chest pain is another frequently observed symptom [13]. 

Gastrointestinal symptoms include nausea, abdominal pain, loss of appetite, or constipation, caused by intestinal dysbiosis, with the virus being present in the gut even 4 months after the onset of the disease [9]. 

### Objectives

The objective of this study is to identify and characterize the prevailing symptoms observed in individuals with the long COVID syndrome and assess their influence on the overall quality of life.

## 2. Materials and Methods

This prospective observational study included 103 patients diagnosed with mild and moderate COVID-19 (based on a positive SARS-CoV-2 molecular diagnostic or rapid antigen test and severity form definition) at the Clinical Hospital of Infectious Diseases, Cluj-Napoca, Romania. 

According to patients’ COVID-19 diagnosis interval and the national data for the circulation of SARS-CoV-2 variants of concern (VOCs), patients were classified as infected with omicron VOC.

Demographic data and information regarding medical history, smoker/non-smoker status, and vaccination status were collected for all the subjects included in this study. 

Clinical examinations with detailed questioning of symptoms and EKG were performed at baseline (the time of the COVID-19 diagnosis) and at the six-month follow-up. Two years after COVID-19 infection, patients were invited to complete an online quality-of-life questionnaire regarding long COVID symptoms.

All the subjects included in this study signed an informed consent form. The study was approved by the Ethics Committee and conducted according to the recommendations of the Declaration of Helsinki for medical research involving human subjects.

## 3. Results

The baseline characteristics of the subjects included in this study are presented in Table 1. A total of 103 patients with a mean age of 41.56 ± 11.77 were included in the study, comprising 35.9% men and 64.1% women. In the entire patient population, 65.04% presented mild forms of COVID-19, while 34.95% exhibited moderate illness severity. No patient included in the study presented severe SARS-CoV-2 infections. Among the subjects, 43.1% were obese/overweight. Regarding smoking status, 25.2% smoked cigarettes. Data regarding the vaccination status showed that 83.5% were vaccinated against SARS-CoV-2. Additionally, one patient suffered from diabetes mellitus, 3.9% had cancer, 16.5% had autoimmune diseases (i.e., Hashimoto thyroiditis), 4.9% had immunosuppressive conditions, 3.9% had chronic kidney disease, 13.6% had chronic lung diseases, and 7.8% had chronic liver diseases, but no patient suffered from cardiac and cerebrovascular diseases.

As shown in Table 1, a significant difference was only found in the relationship between disease severity and age; notably, women who had a moderate form of COVID-19 were older than those with a mild form of the disease (*p* = 0.003). There were no differences regarding disease severity in relation to vaccination status, weight, or comorbidities.

Table 2 displays the symptoms observed at the time of diagnosis, categorized by gender and form of disease.

Headaches were present in 67.6% of the patients, with a higher incidence in female patients (*p* = 0.014). There were no other statistically significant differences found in relation to the other symptoms, disease severity, or gender. 

It was found that respiratory symptoms had a high rate among patients at diagnosis, including rhinorrhea (58.3%), cough (80.6%), odynophagia (76.7%), and smell disorders (22.5%). A minor proportion of patients experienced additional respiratory symptoms, such as dyspnea (20.6%) and hemoptysis (2%). 

The most frequent general symptom was fatigue, found in 80.4% of the patients, followed by fever (45.6%), myalgia (54.9%), and arthralgia (38.2%).

A small percentage of patients presented digestive symptoms like taste disorder, observed in 19.6% of cases; vomiting (6.9%); and diarrhea (11.8%). 

Regarding cardiovascular symptoms at diagnosis (Table 2), the most prevalent symptom was palpitations, occurring in 24.5% of patients, with 23.1% of these being women and 27% men. Chest pain was reported by 11.8% of patients, with 15.4% of women and 5.4% of men experiencing this symptom. 

Additionally, a small percentage of the patients experienced skin rash (4.9%). 

The measurement of blood pressure was performed at diagnosis and during the clinical evaluation at six months (Table 3). The evaluation of blood pressure was performed after 5–10 min rest, in a sitting position; the patients did not smoke or eat or drink 30 min before evaluation. According to statistical analysis, patients who had moderate forms of the disease had higher levels of both systolic (*p* = 0.008) and diastolic blood pressure (*p* = 0.037) at diagnosis, but no statistical difference was observed at the 6-month evaluation. At baseline, there was no significant difference in the mean blood pressure between patients with mild or moderate forms of COVID-19, but after 6 months, a significant difference emerged between the two forms of the disease (*p* = 0.041).

EKG was performed on 91 patients, comprising 60 with a mild form and 31 with a medium form of the disease (Table 4). There were no statistically significant differences observed in the analysis of the amplitude and duration of the P wave, PR interval, QRS duration, RT duration at baseline, and RT duration at 6 months in patients with mild and moderate forms of COVID-19. A statistically significant increase in QTc interval was observed in patients with a mild form of the disease (*p* = 0.031).

Notably, 34 patients received antiviral therapy, 8 of whom had a mild form and 26 had a moderate form of the disease. There was a statistically significant difference observed (*p* < 0.001), with 72.22% of patients with a moderate form receiving antiviral treatment, while only 11.94% of those with a mild form received it.

An online questionnaire was sent to all the patients enrolled in the study to assess their symptoms and quality of life at the 2-year follow-up (complete data being presented in Supplemental Materials). Only 29 subjects filled in the questionnaire. 

We found that the prevalence of ophthalmologic symptoms was significantly more common among female patients (*p* = 0.038). In the other statistical analyses considering the form of the disease, gender, or vaccination status, no significant differences were found. 

At baseline, headaches were observed in 67.6% of patients, but only 34.5% reported it two years later. Regarding other neuropsychiatric symptoms, the most frequent symptoms involved memory disorders in 27.6% of cases, impaired concentration in 24.1% of cases, insomnia in 24.1% of patients, and paraesthesia in 20.7% of cases. Overall, 17.2% of patients reported attention deficit disorder at 2 years, and 13.8% reported increased anxiety, while only 3.4% of patients reported tremors and cognitive impairment. Balance disorder and vertigo were reported in 20.7% of cases, followed by tinnitus in 10.3% of cases, and hearing loss in 7.1% of cases.

Regarding skin modifications, 13.8% of the patients reported hair loss, and only 10.3% reported nail modification and skin rash.

Asthenia and decreased effort tolerance were reported by more than half of patients (51.7%), followed by fatigue (20.7%) and dyspnea (10.3%).

The reported respiratory symptoms included dry cough (20.7%), productive cough (17.2%), rhinorrhea (10.3%), and nasal congestion (24.1%). In 20.7% of patients, dysphonia and odynophagia were observed to equal degrees.

About a quarter of patients experienced abdominal meteorism, which was the most reported digestive symptom (27.6%), followed by abdominal pain and weight gain, which were recorded in 17.2% of cases. Intestinal transit disorders such as diarrhea (3.4%) and constipation (13.8%) were also reported. Less than 15% of patients reported nausea and vomiting (13.8%), hyposmia (10.3%), digestive intolerance and dysgeusia (6.9%), and oral thrush (3.4%).

Regarding osteoarticular symptoms, arthralgia (27.6%) and myalgia (20.7%) were reported by almost a quarter of patients. Generalized pain was diagnosed in 13.8% of cases, and joint swelling was diagnosed in 3.4% of patients.

Menstrual disorders were recorded in 17.2% of female patients.

**Figure 1:**
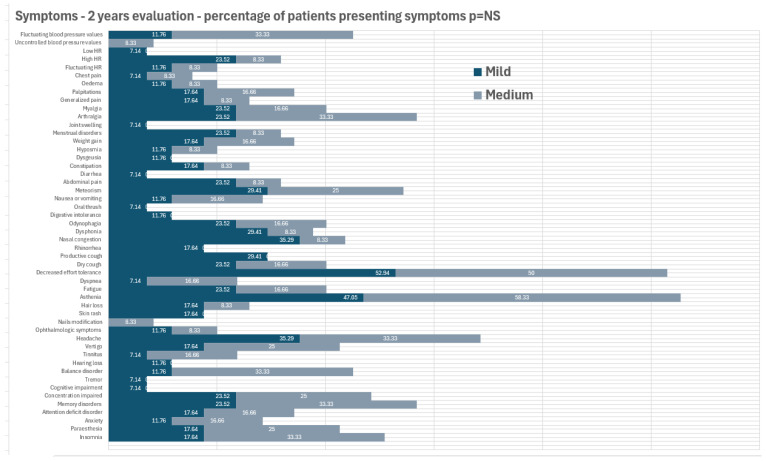
Symptoms at 2-year follow-up in relation to disease severity.

The most common cardiovascular symptoms were fluctuating blood pressure values (20.7%), palpitations (17.2%), and increased heart rate (17.2%). Edema and fluctuating heart rates were reported by 10.3% of the patients, followed by chest pain, recorded in 6.9% of the cases. Occasionally, individuals experienced bradycardia and hypertension, with a prevalence of 3.4% for each condition. No significant differences were observed regarding the symptoms’ presence between males and females (with the only exception being ophthalmologic symptoms, which were more prevalent in women (0% vs. 33% *p* = 0.038)). No significant differences were found in the relationship between disease severity and vaccinal status—as it is presented in Figure 1. Complete data are provided in Supplemental Materials.

Considering 29 patients, a comparison was performed between symptoms’ presence at baseline vs. 2-year follow-up (using concordance coefficient); the registered kappa values were as follows: headache—0.166, cough—0.0962, rhinorrhea—0.044, odynophagia—0.0973, myalgia—0.0443, arthralgia—0.086, dyspnea—0.208, nausea or vomiting—0.0584, diarrhea—0.0357, fatigue—0.125, palpitations—0.208, and chest pain—0.346.

## 4. Discussion

The COVID-19 pandemic is still a burden for healthcare systems worldwide. The current concern is not only related to the acute forms of COVID-19 but also the long-term effects of COVID-19 infection, defined as long COVID [3]. Patients remain symptomatic for extended periods following the COVID-19 diagnosis [14] and may even trigger other conditions, such as hypertension [15], orthostatic tachycardia syndrome, cerebrovascular diseases, and thromboembolic events [9]. Besides cardiovascular comorbidities, new onset of type 2 diabetes mellitus, myalgic encephalomyelitis, and chronic fatigue syndrome are also encountered among patients with long COVID [9].

Our study focused on mild and moderate COVID-19 cases. Women who had a moderate form of COVID-19 were older than those with a mild form of the disease, and there was no other statistically significant correlation between disease severity and the vaccination status, weight, or patients’ comorbidities. However, some studies have found no sex differences regarding the COVID-19 diagnosis in the general population [16]. Contrarily, COVID-19-associated death was higher in women with comorbidities such as heart failure and dementia [17,18], but the overall mortality was higher in males [19].

At diagnosis, the most frequent symptoms were cough, odynophagia, headaches, and fatigue. Women were more likely to experience cephalalgia than men. A higher frequency of headaches among female patients was also found in a study conducted on 2194 patients by García-Azorín et al. [20]. As for the most frequent symptoms experienced by the subjects included in our study, cough was present in 80.6% of patients and fatigue in 80.4% at the time of diagnosis, while the main symptoms of COVID-19 in the literature were fever and cough [21,22,23].

The new onset of hypertension, both systolic and diastolic, following SARS-CoV-2 infection was identified in several studies [1,24], with women being more frequently affected [24]. In our study, patients who had moderate forms of the disease had higher levels of both systolic and diastolic blood pressure at diagnosis. Additionally, some patients with no cardiovascular diseases prior to SARS-CoV-2 infection developed prehypertension.

Regarding the ECG at the time of diagnosis, a statistically significant increase in QTc interval was identified among patients with mild COVID-19. Pornwattanakavee et al. found that QTc prolongation was present in patients with comorbidities that represent the risk factors for this ECG modification, and it was encountered after treatment initiation, which could also contribute to its development [25].

An online questionnaire was completed by patients during their 2-year reassessment in our study. The most common symptoms present at the 2-year re-evaluation were asthenia, decreased effort tolerance, insomnia, memory disorders, headache, concentration impairment, vertigo, paresthesia, vertigo, and fatigue. Additionally, a notable proportion of patients presented persistent cough, dysphonia, and odynophagia. 

No significant correlation was found between symptoms’ presence at the time of diagnosis vs. 2-year follow-up, with the exception of cardiovascular symptoms like dyspnea, palpitation, and chest pain (a kappa coefficient of 0.2–0.4 shows a fairly strong agreement). The most striking observation from the analysis was the fact that initial symptoms did not persist over time, and patients were capable of developing new ones; these results provide support for continuous patient evaluation and observation. The prevalence of ophthalmologic symptoms was significantly higher among female patients. This could be the result of nerve injury, namely the corneal nerve injury, together with an increase in the density of the dendritic cells of the cornea [26,27]. Studies show that the quality of life in patients with visual impairment is poor [28,29], which is worse than that of patients with diabetes mellitus type 2 [30], coronary artery disease, and hearing disorders [28].

### Limitations of the Study

The most important limitation is that the number of participants at the 2-year follow-up was very low (29 subjects), as patients were not interested in filing in the follow-up form, which partially limited the validity of the conclusions. Also, at the time of evaluation, no data regarding previous infections were collected. At the same time, we have to mention the fact that patients’ classification as infected with SARS-CoV-2 omicron VOC was based on national data on the predominance of VOCs [31], but the different VOCs co-circulated, and no individual identification was available, which limited the absolute association with the omicron variant. 

## 5. Conclusions

In conclusion, our study not only presents a comprehensive summary of the symptoms and clinical changes present in patients with mild and moderate forms of COVID-19 (omicron variant) at the time of diagnosis but also involves the analysis of their persistence over 6 months and 24 months. Thus, if at diagnosis the most frequent manifestations of the disease were respiratory, together with headache and fatigue, at the time of re-evaluation, asthenia, decreased effort tolerance, and neuropsychiatric symptoms prevailed. 

Our data reveal that the probability of having long COVID is high in symptomatic forms and can affect the quality of life, so it is essential to monitor patients even after a negative swab. 

Regarding cardiovascular changes as part of the clinical picture of long COVID, some patients developed prehypertension, palpitations, and tachycardia.

## Figures and Tables

**Table 1:** Baseline characteristics of the included patients in relation to the severity of the disease and gender.

		Global			Male			Female		
		Mild	Moderate	*p*-Value	Mild	Moderate	*p*-Value	Mild	Moderate	*p*-Value
Number of Patients		67	36		21	16		46	20	
Age		38.89 ± 11.56	46.52 ± 10.61	0.0014	42.33 ± 10.68	46.43 ± 11.83	0.2766	37.32 ± 11.72	46.6 ± 9.84	0.003
Obesity/overweight	Yes	25 (37.31)	19 (52.77)	0.1520	9 (42.85)	10 (62.5)	0.3940	16 (34.78)	9 (45)	0.5039
	No	42 (62.68)	17 (47.22)		12 (57.14)	6 (37.5)		30 (65.21)	11 (55)	
Smoking	Yes	18 (26.86)	8 (22.22)	0.7799	6 (28.57)	1 (6.25)	0.1957	12 (26.08)	7 (35)	0.6605
	No	49 (73.13)	28 (77.77)		15 (71.42)	15 (93.75)		34 (73.91)	13 (65)	
Diabetes	Yes	1 (1.49)	0 (0)	0.7511	1 (4.76)	0 (0)	0.8900	0 (0)	0 (0)	NS
	No	66 (98.50)	36 (100)		20 (95.23)	16 (100)		46 (100)	20 (100)	
Pulmonary disease	Yes	8 (11.94)	6 (16.66)	0.7144	1 (4.76)	3 (18.75)	0.4104	7 (15.21)	3 (15)	0.7257
	No	59 (88.05)	30 (83.33)		20 (95.23)	13 (81.25)		39 (84.78)	17 (85)	
Vaccinated	Yes	56 (83.58)	30 (83.33)	0.8058	18 (85.71)	13 (81.25)	0.9321	38 (82.6)	17 (85)	0.9047
	No	11 (16.41)	6 (16.66)		3 (14.28)	3 (18.75)		8 (17.39)	3 (15)	

**Table 2:** The symptoms of COVID-19 at diagnosis.

Positive Symptoms	Global	Mild	Moderate	*p* Low vs. Moderate	Male Global	Mild	Moderate	*p* Low vs. Moderate	Female Global	Mild	Moderate	*p* Low vs. Moderate	*p* Male vs. Female
Rhinorrhea	60 (58.3)	41 (61.2)	19 (52.8)	0.537	25 (67.6)	14 (66.7)	11 (68.7)	0.825	35 (53)	27 (58.7)	8 (40)	0.258	0.219
Cough	83 (80.6)	53 (79.1)	30 (83.3)	0.797	30 (81.1)	16 (76.2)	14 (87.5)	0.655	53 (80.3)	37 (80.4)	16 (80)	0.767	0.869
Odynophagia	79 (76.7)	52 (77.6)	27 (75)	0.956	27 (73)	15 (71.4)	12 (75)	0.895	52 (78.8)	37 (80.4)	15 (75)	0.866	0.669
Smell disorder	23 (22.5)	14 (21.2)	9 (25)	0.849	8 (21.6)	3 (14.3)	5 (31.2)	0.401	15 (23.1)	11 (24.4)	4 (20)	0.941	0.938
Dyspnea	21 (20.6)	15 (22.7)	6 (16.7)	0.640	4 (10.8)	2 (9.5)	2 (12.5)	0.806	17 (26.2)	13 (28.9)	4 (20.0)	0.655	0.112
Hemoptysis	2 (2.0)	2(3)	-	0.758	-	-	-	-	2 (3.1)	2 (4.4)		0.857	0.737
Fever	47 (45.6)	31 (46.3)	16 (44.4)	0.975	18 (48.6)	12 (57.1)	6 (37.5)	0.394	29 (43.9)	19 (41.3)	10 (50)	0.700	0.799
Headaches	69 (67.6)	45 (68.2)	24 (66.7)	0.948	19 (51.4)	11(52.4)	8 (50)	0.850	50 (76.9)	34 (75.6)	16 (80)	0.941	0.014
Myalgia	56 (54.9)	38 (57.6)	18 (50)	0.598	18 (48.6)	10 (47.6)	8 (50)	0.850	38 (58.5)	28 (62.2)	10 (50)	0.515	0.452
Arthralgia	39 (38.2)	27 (40.9)	12 (33.3)	0.589	10 (27.0)	7 (33.3)	3 (18.8)	0.537	29 (44.6)	20 (44.4)	9 (45.0)	0.819	0.122
Skin rash	5 (4.9)	3 (4.5)	2 (5.6)	0.799	2 (5.4)	-	2 (12.5)	0.351	3 (4.6)	3 (6.7)	-	0.587	0.764
Taste disorder	20 (19.6)	12 (18.2)	8 (22.2)	0.817	7 (18.9)	2 (9.5)	5 (31.2)	0.212	13 (20)	10 (22.2)	3 (15)	0.736	0.898
Vomiting	7 (6.9)	7 (10.6)	-	0.106	2 (5.4)	2 (9.5)	-	0.592	5 (7.7)	5 (11.1)	-	0.295	0.974
Diarrhea	12 (11.8)	9 (13.6)	3 (8.3)	0.636	6 (16.2)	4 (19.0)	2 (12.5)	0.932	6 (9.2)	5 (11.1)	1 (5.0)	0.747	0.463
Fatigue	82 (80.4)	55 (83.3)	27 (75.0)	0.452	28 (75.7)	15 (71.4)	13 (81.2)	0.761	54 (83.1)	40 (88.9)	14 (70.0)	0.129	0.518
Palpitations	25 (24.5)	16 (24.2)	9 (25.0)	0.876	10 (27.0)	5 (23.8)	5 (31.2)	0.895	15 (23.1)	11 (24.4)	4 (20.0)	0.941	0.836
Chest pain	12 (11.8)	10 (15.2)	2 (5.6)	0.265	2 (5.4)	1 (4.8)	1 (6.2)	0.592	10 (15.4)	9 (20)	1 (5.0)	0.240	0.236

**Table 3:** The impact of COVID on blood pressure values at baseline and at 6-month evaluation.

	Mild (n = 51)	Medium (n = 28)	Difference (95% CI)	*p*-Value
Mean arterial pressure (mmHg) at diagnosis, median	81 (76–87)	86 (78–90)	5 (−8–0)	0.054
Mean arterial pressure (mmHg) at 6 months, median	83 (76–89)	88 (80–99)	5 (−15–0)	0.041
Systolic BP at diagnosis (mmHg), median	114 (99.25–126)	120 (113.5–134.5)	6 (−16–−2)	0.008
Systolic BP (mmHg) 6 months, median	117 (110–121)	125 (111–135)	8 (−19–1)	0.086
Diastolic BP (mmHg) at diagnosis, median	64 (60–70)	69 (63–73)	5 (−7–0)	0.037
Diastolic BP (mmHg) 6 months, median	66 (59–74)	70 (66–80)	4 (−13–1)	0.073

**Table 4:** EKG modifications at diagnosis.

EKG	Mild (n = 60)	Medium (n = 31)	Difference (95% CI)	*p*-Value
P wave amplitude (mV) baseline, median	0.1 (0.1–0.2)	0.1 (0.1–0.15)	0 (0–0)	0.353
P wave duration (ms) baseline, median	100 (80–100)	80 (80–100)	20 (0–10)	0.169
PR interval duration (ms) baseline, median	140 (140–160)	160 (140–160)	20 (−20–0)	0.068
QRS duration (ms) baseline, median	95 (90–100)	90 (85–100)	5 (0–10)	0.117
QTc (ms) baseline, median	402 (380.5–416)	388 (371.5–402.5)	14 (1–26)	0.031
RT (ms) baseline, median	133 (121–141)	137 (115–145)	4 (−10–9)	0.704
RT (ms) 6 months, median	135 (121–147)	123 (111–138)	12 (−5–21)	0.24

## Data Availability

The data that support the findings of this study are available upon request from the corresponding author. The data are not publicly available due to privacy or ethical restrictions.

## Supplementary Materials

The following supporting information can be downloaded at: https://www.mdpi.com/article/10.3390/medicina60081359/s1.
