# Supplementary material for: The Impact of Long COVID on the Quality of Life
Source: Medicina (Kaunas). 2024 Aug 21;60(8):1359. doi: 10.3390/medicina60081359 (PMC11356039; doi:10.3390/medicina60081359)
Supplement: Supplementary file 1 [file medicina-60-01359-s001.zip › medicina-3161843-supplementary.pdf]

Supplemental material

## Symptoms at 2 years evaluation

| Symptoms - yes | Global | Mild | Medium | ***P* low-medium** | Female Global | Male Global | ***P* Female-male** | No vaccinated | Vaccinated | ***P* Vaccinated-non vaccinated** |
|---|---|---|---|---|---|---|---|---|---|---|
| | No (%) 29(100) | No (%) 17(100) | No (%) 12(100) | | No (%) 20(100) | No (%) 9 (100) | | No (%) 4(100) | No (%) 25(100) | |
| Insomnia | 7 (24.1%) | 3 (17.64) | 4(33.33) | 0.594 | 5(25) | 2(22.22) | 0.758 | 0(0) | 7(28) | 0.558 |
| Paraesthesia | 6 (20.7%) | 3(17.64) | 3 (25) | 0.987 | 5(25) | 1(11.11) | 0.719 | 2 (50) | 4 (16) | 0.371 |
| Anxiety | 4 (13.8%) | 2 (11.76) | 2(16.66) | 0.865 | 4(20) | 0(0) | 0.388 | 0(0) | 4(16) | 0.935 |
| Attention deficit disorder | 5 (17.2%) | 3 (17.64) | 2(16.66) | 0.667 | 5(25) | 0(0) | 0.263 | 1(25) | 4(16) | 0.786 |
| Memory disorders | 8 (27.6%) | 4 (23.52) | 4(33.33) | 0.872 | 5(25) | 3(33.33) | 0.987 | 3(75) | 5(20) | 0.092 |
| Concentration impaired | 7 (24.1%) | 4(23.52) | 3(25) | 0.726 | 6 (30) | 1(11.11) | 0.528 | 1(25) | 6(24) | 0.558 |
| Cognitive impairment | 1 (3.4%) | 1 (7.14) | 0(0) | 0.858 | 1 (5) | 0(0) | 0.676 | 0(0) | 1 (4) | 0.285 |
| Tremor | 1 (3.4%) | 1 (7.14) | 0(0) | 0.858 | 1(5) | 0(0) | 0.676 | 0(0) | 1(4) | 0.285 |
| Balance disorder | 6 (20.7%) | 2 (11.76) | 4(33.33) | 0.343 | 4(20) | 2(22.22) | 0.719 | 1(25) | 5(20) | 0.663 |
| Hearing loss | 2 (7.1%) | 2(11.76) | 0(0) | 0.596 | 2(10) | 0(0) | 0.822 | 1(25) | 1(4) | 0.497 |
| Tinnitus | 3 (10.3%) | 1 (7.14) | 2(16.66) | 0.748 | 2(10) | 1(11.11) | 0.570 | 0(0) | 3(12) | 0.878 |
| Vertigo | 6 (20.7%) | 3 (17.64) | 3(25) | 0.987 | 6(30) | 0(0) | 0.177 | 1(25) | 5(20) | 0.663 |
| Headache | 10(34.5%) | 6 (35.29) | 4(33.33) | 0.774 | 7 (35) | 3(33.33) | 0.737 | 1(25) | 9(36) | 0.891 |
| Ophthalmologic symptoms | 3 (10.3%) | 2 (11.76) | 1(8.33) | 0.748 | 0(0) | 3(33.33) | **0.038** | 0(0) | 3 (12) | 0.878 |
| Nails modification | 3 (10.3%) | 2 (11.76) | 1(8.33) | 0.748 | 3(15) | 0(0) | 0.570 | 1(25) | 2(8) | 0.878 |
| Skin rash | 3 (10.3%) | 3 (17.64) | 0(0) | 0.358 | 3(15) | 0(0) | 0.570 | 1(25) | 2(8) | 0.878 |
| Hair loss | 4 (13.8%) | 3 (17.64) | 1(8.33) | 0.865 | 3(15) | 1(11.11) | 0.763 | 1(25) | 3(12) | 0.935 |
| Asthenia | 15(51.7%) | 8 (47.05) | 7(58.33) | 0.825 | 12 | 3(33.33) | 0.353 | 1(25) | 14(56) | 0.539 |
| Fatigue | 6 (20.7%) | 4 (23.52) | 2(16.66) | 0.987 | 4(20) | 2(22.22) | 0.719 | 1(25) | 5(20) | 0.663 |
| Dyspnea | 3 (10.3%) | 1 (7.14) | 2(16.66) | 0.748 | 3(15) | 0(0) | 0.570 | 0(0) | 3(12) | 0.878 |
| Decreased effort tolerance | 15 (51.7%) | 9 (52.94) | 6 (50) | 0.825 | 11(55) | 4(44.44) | 0.900 | 1(25) | 14(56) | 0.539 |
| Dry cough | 6 (20.7%) | 4 (23.52) | 2(16.66) | 0.987 | 4(20) | 2(22.22) | 0.719 | 0(0) | 6(24) | 0.663 |
| Productive cough | 5 (17.2%) | 5 (29.41) | 0(0) | 0.117 | 4(20) | 1(11.11) | 0.956 | 1(25) | 4(16) | 0.786 |
| Rhinorrhea | 3 (10.3%) | 3 (17.64) | 0(0) | 0.358 | 3(15) | 0(0) | 0.570 | 0(0) | 3(12) | 0.878 |

| | | | | | | | | | | | | |
|---|---|---|---|---|---|---|---|---|---|---|---|---|
| Nasal congestion | 7 (24.1%) | 6 (35.29) | 1(8.33) | 0.218 | | 6(30) | 1(11.11) | 0.528 | | 1(25) | 6(24) | 0.558 |
| Dysphonia | 6 (20.7%) | 5 (29.41) | 1(8.33) | 0.360 | | 5(25) | 1(11.11) | 0.719 | | 1(25) | 5(20) | 0.663 |
| Odynophagia | 6 (20.7%) | 4 (23.52) | 2(16.66) | 0.987 | | 4(20) | 2(22.22) | 0.719 | | 1(25) | 5(20) | 0.663 |
| Digestive intolerance | 2 (6.9%) | 2 (11.76) | 0(0) | 0.626 | | 2(10) | 0(0) | 0.848 | | 0(0) | 2(8) | 0.633 |
| Oral thrush | 1 (3.4%) | 1 (7.14) | 0(0) | 0.858 | | 1(5) | 0(0) | 0.676 | | 0(0) | 1(4) | 0.285 |
| Nausea or vomiting | 4 (13.8%) | 2 (11.76) | 2(16.66) | 0.865 | | 3(15) | 1(11.11) | 0.763 | | 0(0) | 4(16) | 0.935 |
| Meteorism | 8 (27.6%) | 5 (29.41) | 3(25) | 0.872 | | 7(35) | 1(11.11) | 0.377 | | 1(25) | 7(28) | 0.632 |
| Abdominal pain | 5 (17.2%) | 4 (23.52) | 1(8.33) | 0.570 | | 5(25) | 0(0) | 0.263 | | 1(25) | 4(16) | 0.786 |
| Diarrhea | 1 (3.4%) | 1 (7.14) | 0(0) | 0.858 | | 1(5) | 0(0) | 0.676 | | 1(25) | 0(0) | 0.285 |
| Constipation | 4 (13.8%) | 3 (17.64) | 1(8.33) | 0.865 | | 4(20) | 0(0) | 0.388 | | 1(25) | 3(12) | 0.935 |
| Dysgeusia | 2 (6.9%) | 2 (11.76) | 0(0) | 0.626 | | 2(10) | 0(0) | 0.848 | | 1(25) | 1(4) | 0.633 |
| Hyposmia | 3 (10.3%) | 2 (11.76) | 1(8.33) | 0.748 | | 3(15) | 0(0) | 0.570 | | 1(25) | 2(8) | 0.878 |
| Weight gain | 5 (17.2%) | 3 (17.64) | 2(16.66) | 0.667 | | 4(20) | 1(11.11) | 0.956 | | 1(25) | 4(16) | 0.786 |
| Menstrual disorders | 5 (17.2%) | 4 (23.52) | 1(8.33) | 0.570 | | 5(25) | 0 (0) | - | | 1(25) | 4(16) | 0.786 |
| Joint swelling | 1 (3.4%) | 1 (7.14) | 0(0) | 0.858 | | 1(5) | 0(0) | 0.676 | | 1(25) | 0(0) | 0.285 |
| Arthralgia | 8 (27.6%) | 4 (23.52) | 4(33.33) | 0.872 | | 7(35) | 1(11.11) | 0.377 | | 1(25) | 7(28) | 0.632 |
| Myalgia | 6 (20.7%) | 4 (23.52) | 2(16.66) | 0.987 | | 5(25) | 1(11.11) | 0.719 | | 2(50) | 4(16) | 0.371 |
| Generalized pain | 4 (13.8%) | 3 (17.64) | 1(8.33) | 0.865 | | 4(20) | 0(0) | 0.388 | | 1(25) | 3(12) | 0.935 |
| Palpitations | 5 (17.2%) | 3 (17.64) | 2(16.66) | 0.667 | | 4(20) | 1(11.11) | 0.956 | | 0(0) | 5(20) | 0.786 |
| Oedema | 3 (10.3%) | 2 (11.76) | 1(8.33) | 0.748 | | 3(15) | 0(0) | 0.570 | | 1(25) | 2(8) | 0.878 |
| Chest pain | 2 ( 6.9%) | 1 (7.14) | 1(8.33) | 0.626 | | 2(10) | 0(0) | 0.848 | | 0(0) | 2(8) | 0.633 |
| Fluctuating HR | 3 (10.3%) | 2 (11.76) | 1(8.33) | 0.748 | | 2(10) | 1(11.11) | 0.570 | | 1(25) | 2(8) | 0.878 |
| High HR | 5 (17.2%) | 4 (23.52) | 1(8.33) | 0.570 | | 4(20) | 1(11.11) | 0.956 | | 0(0) | 5(20) | 0.786 |
| Low HR | 1 (3.4%) | 1 (7.14) | 0(0) | 0.858 | | 1(5) | 0(0) | 0.676 | | 0(0) | 1(4) | 0.285 |
| Uncontrolled blood pressure values | 1 (3.4%) | 0 (0) | 1(8.33) | 0.858 | | 0(0) | 1(11.11) | 0.676 | | 0(0) | 1(4) | 0.285 |
| Fluctuating blood pressure values | 6 (20.7%) | 2 (11.76) | 4(33.33) | 0.343 | | 4(20) | 2(22.22) | 0.719 | | 0(0) | 6(24) | 0.663 |
